# Endotoxin Triggers Tumor Initiation Events in Nontumorigenic Breast Epithelial Cells and Enhances Invasion-Related Phenotype in Pretumorigenic and Tumorigenic Breast Epithelial Cells

**DOI:** 10.1155/2021/4666380

**Published:** 2021-11-26

**Authors:** Farah Yassine, Sabreen F. Fostok, Nataly Naser Al Deen, Rabih S. Talhouk

**Affiliations:** American University of Beirut (AUB), Department of Biology, Beirut, Lebanon

## Abstract

Inflammation is associated with the development of several cancers, including breast cancer. However, the molecular mechanisms driving breast cancer initiation or enhancement by inflammation are yet to be deciphered. Hence, we opted to investigate the role of inflammation in initiating and enhancing tumor-like phenotypes in nontumorigenic, pretumorigenic, and tumorigenic breast epithelial cells. Noncytotoxic endotoxin (ET) concentrations capable of inducing an inflammatory phenotype were determined for the different cell lines. Results showed that short-term ET exposure upregulated matrix metalloproteinase-9 (MMP-9) activity in nontumorigenic mammary epithelial cells of mouse (SCp2) and human origins (HMT-3522 S1; S1) and upregulated inflammatory mediators including nitric oxide (NO) and interleukin 1-*β* in tumorigenic human breast cells (MDA-MB-231), all in a dose-dependent manner. Long-term ET treatment, but not short-term, triggered the migration of SCp2 cells, and proliferation and migration of tumorigenic human breast cells MCF-7 and MDA-MB-231. Both short- and long-term ET exposures preferentially enhanced the invasion of pretumorigenic S1-connexin 43 knockout (Cx43-KO S1) cells compared to their nontumorigenic S1 counterparts. Moreover, both ET exposures disrupted lumen formation and apicolateral distribution of *β*-catenin in 3D cultures of S1 cells. In conclusion, ET treatment at concentrations that elicited inflammatory phenotype triggered tumor initiation events in nontumorigenic and pretumorigenic breast cells, and increased tumorigenicity of breast cancer cells. Our findings highlight the role of inflammation in enhancing migration, invasion, and loss of normal 3D morphology and suggest that such inflammatory insults can “add injury” to pretumorigenic and tumorigenic breast epithelial cells.

## 1. Introduction

Inflammation is implicated in the initiation processes of several cancers [1]. Specifically, chronic inflammation has long been associated with cancer development [2]. For instance, the risk of developing colorectal cancer increases with inflammatory bowel diseases such as ulcerative colitis and Crohn's disease [3, 4]. The risk of hepatocellular carcinoma is also more pronounced in the setting of chronic hepatitis and cirrhosis [5]. Similarly, gastric inflammation caused by *Helicobacter pylori* infection has been linked to gastric malignancies [6].

Endotoxin (ET), a lipopolysaccharide constituent of Gram-negative bacterial cell wall, is a known inflammatory trigger implicated in cancer invasion and angiogenesis [7–9]. Chronic exposure to ET was shown to promote lung tumorigenesis [10], and by affecting key determinants of metastasis, ET was shown to enhance migration of prostate cancer cells [11].

Among all cancer types, breast cancer is the most common and the second cause of cancer-related deaths in women after lung cancer worldwide [12]. A complex relationship has been described between inflammation and breast cancer risk [13]. For example, elevated levels of C-reactive protein, an inflammatory marker, were associated with an increase in the risk of breast cancer [14]. However, the association between chronic inflammation and transition of the normal breast epithelium into neoplastic tissue is not well understood. Studies showed the involvement of the inflammatory microenvironment in the malignant progression of breast cancer [15]. Thus, studying the inflammatory biomarkers, cellular mediators, and their downstream effects due to a chronic insult is important for understanding cancer initiation [16].

In light of the above, little is known about the effect of ET-induced chronic inflammation on breast cancer initiation events and whether such inflammation would trigger loss of 3D morphological differentiation and apical polarity that characterize normal breast tissue.

Previous studies by our laboratory showed that ET-activated NF-kB suppressed *β*-casein expression and upregulated gelatinases, cytokines, nerve growth factor (NGF), and nitric oxide (NO) in rodent mammary cells [17, 18]. Thus, we opted to study the effect of short- and long-term ET inflammatory challenge in nontumorigenic, pretumorigenic, and tumorigenic human breast epithelial cell lines. We propose that ET-induced inflammation enhances the tumor initiation events in nontumorigenic and pretumorigenic mammary epithelial cells and increases tumorigenicity of breast cancer cell lines, suggesting that inflammatory insults not only trigger tumor initiation events but can also “add injury” to pretumorigenic and tumorigenic breast cells.

## 2. Materials and Methods

### 2.1. Cell Culture

For the nontumorigenic mouse mammary epithelial cell (SCp2) culture, low passage numbers (18–25) of SCp2 cells were used throughout. Cells (kindly provided by P. Y. Desprez, Geraldine Brush Cancer Research Institute, California Pacific Medical Center, San Francisco, CA) were maintained and propagated as described by Maalouf et al. [17].

For nontumorigenic human breast cells HMT-3522 S1 (S1) and pretumorigenic S1-connexin 43 knockout (Cx43-KO S1) cultures, methods by Fostok et al. [19] were followed for both two-dimensional (2D) and 3D conditions. Cells were kindly provided by Dr. Sophie Lelievre (Purdue University, IN).

For tumorigenic human breast cells MDA-MB-231 and MCF-7 cultures, kindly provided by Dr. Mina Bissell (Lawrence Berkeley National Laboratory, LBNL, CA), low passage numbers (18–30) were grown in humidified incubator (95% air, 5% CO2) at 37°C, in RPMI 1640 media (Lonza, Belgium) supplemented with 1% penicillin-streptomycin (Pen-Strep) and 10% fetal bovine serum (FBS). The medium was changed once every two days, and cells were transferred at 80% confluence.

Short-term ET exposure consisted of ET treatment for 48 hours after acquisition of 70–80% confluence, after which conditioned media were collected and assayed, whereas long-term exposure consisted of a continuous ET treatment for a one-month period of time, replenished with every change of media, in an attempt to mimic chronic inflammation. For all ET treatments, ET was added at concentrations (0.1–10 *µ*g/ml) that did not affect cell viability while eliciting an inflammatory response.

### 2.2. Zymography Assay (Substrate-Gel Electrophoresis)

Culture media were collected from the respective cultures and stored at −80°C. Gelatinase activity in the collected media was analyzed using the method described by Talhouk et al. [20]. Equal sample volumes mixed in 1 : 1 ratio (V/V) with 2X sample buffer were loaded and run on 7% polyacrylamide gels impregnated with gelatin (4.5 mg/ml). The gelatinases appeared as clear white bands on darkly stained blue gels; then, colors were inverted using ImageJ (http://imagej.nih.gov/ij/) software in order to visualize the gelatinases as black bands against a white background as presented. Peak areas of MMP bands were quantified using ImageJ in triplicate, and data were represented as the average fold increase of MMP band peak area (Arbitrary Basal Density) of three independent experiments ± standard deviation (SD) (AU ± SD).

### 2.3. Wound Healing Assay

Untreated cells and those subjected to long-term ET treatment were plated in 6-well plates at a density of 2.5 × 10^5^ cells/ml in their respective culture media (DMEM/F12 containing 1% Pen-Strep, 5% FBS, and 0.1% insulin for SCp2 cells and RPMI 1640 supplemented with 1% Pen-Strep and 10% FBS for MDA-MB-231 and MCF-7 cells). Culture media of cells subjected to long-term ET treatment were also supplemented with 0.1 *µ*g/ml ET for SCp2 cells and 1 *µ*g/ml ET for MDA-MB-231 and MCF-7 cells. After 72 hours, upon reaching full confluence, cells were washed twice with phosphate buffered saline (PBS) 1X and then supplemented with growth media containing 1% Pen-Strep and 1% FBS. Control cells were left untreated, and another group was subjected to short-term ET exposure by supplementing their culture media with 0.1 *µ*g/ml of ET for SCp2 cells and 1 *µ*g/ml ET for MDA-MB-231 and MCF-7 cells. Culture media of cells subjected to long-term ET treatment were also supplemented with ET as before. At the same time, a straight wound was made using a 200 *µ*l pipette tip, and the wound site was monitored throughout time at 2, 6, 12, and 48 hours after wounding. Pictures were taken using a light microscope, and the closure of the wounded area was measured 48 hours after wounding using ImageJ.

### 2.4. Invasion Assay

As reported by Fostok et al. [19], 6-well tissue-culture plates were fitted with inserts (8 *μ*m pore size). The inserts were coated with 400 *μ*l of [Matrigel] Engelbreth-Holm-Swarm (EHS) growth media solution of 1 : 20 ratio and incubated at 37°C for 4 hours; 3 × 10^5^ S1 cells were seeded in the inserts. After 24 hours, the cells were fixed using 4% formaldehyde in PBS 1X for 20 minutes at room temperature. The cells towards the inside of the insert were removed by using a cotton swab, and nuclei of migrated cells were counterstained with Hoechst 33342 (Molecular Probes, H3570) at a concentration of 0.5 *μ*g/ml for 10 minutes at room temperature. The insert was then cut and mounted on a microscopic slide in ProLong® Gold antifade reagent (Invitrogen Molecular Probes). The inserts were then examined by fluorescence microscopy.

### 2.5. Immunofluorescence Labeling

S1 acini from fresh 3D cultures on day 11 were permeabilized with 0.5% peroxide and carbonyl-free Triton X-100 (Sigma-Aldrich) in cytoskeleton buffer (100 mM NaCl, 300 mM sucrose, 10 mM PIPES, pH 6.8, 5 mM MgCl_2_, 1 mM Pefabloc, 10 *µ*g/ml aprotinin, 250 *µ*M NaF) prior to fixation in 4% formaldehyde (Sigma-Aldrich). Antibodies used were rabbit polyclonal *β*-catenin (1 : 100, Santa Cruz Biotechnology, 200 *µ*g/ml). Donkey anti-rabbit secondary antibody conjugated to Alexa Fluor 568 (red) (Invitrogen Molecular Probes, Eugene, OR) was used at the manufacturer's proposed dilution (1 : 2,000). Nuclei were counterstained with 0.5 *µ*g/ml Hoechst 33342 (Molecular Probes, H3570), and specimens were mounted in ProLong® Gold antifade reagent (Invitrogen Molecular Probes). A minimum of one hundred acini were analyzed for each immunostaining using laser scanning confocal microscope (LSCM). Images of immunofluorescence labeling were recorded using LSCM (LSM 410, Zeiss, Germany). Images were processed using ZEN lite software and ImageJ and assembled using Adobe Photoshop® 6.0 (Adobe Systems, San Jose, CA).

### 2.6. Griess Reaction Assay of Nitric Oxide for Nitric Oxide Synthase Activity

Untreated MDA-MB-231 cells were plated in 6-well plates at a density of 2.5 × 10^5^ cells/ml in RPMI 1640 culture media supplemented with 1% Pen-Strep and 10% FBS. After 24 hours, cells were washed twice with PBS 1X and then supplemented with RPMI 1640 containing 1% Pen-Strep and 1% FBS. MDA-MB-231 cells were either left as control untreated cells or subjected to short-term ET exposure by supplementing their culture media with 0.1 or 1 *µ*g/ml ET. Conditioned media were collected 48 hours after treatment [17].

The analysis of NO was done by the Griess assay that measures nitrite (the stable spontaneous oxidation product of NO) using a Griess Reagent Kit (Molecular Probes, Eugene, OR) according to the manufacturer's protocol. Samples were assayed in duplicate, and data were represented as the average concentration of NO_2_^−^ of three independent experiments ± SD (*μ*g ± SD).

### 2.7. Enzyme-Linked Immunosorbent Assay

To measure interleukin 1-*β* (IL-1*β*) secretion in response to ET in MDA-MB-231 cells, media collected 48 hours after ET treatment (as described above) were assayed by enzyme-linked immunosorbent assay (ELISA) for IL-1*β* (DuoSet Kit; R&D Systems Inc., Minneapolis, MN) according to the manufacturer's protocol. Samples were assayed in duplicate, and data were represented as the average IL-1*β* (pg)/10^6^ cells of three independent experiments ± SD.

### 2.8. Statistical Analysis

Data were presented as means ± SD, and statistical comparisons were done using Microsoft Excel. Unpaired or paired *t*-test was used for comparison of two groups, whereas one-way analysis of variance (ANOVA) with Tukey test was employed for three or more groups of treatment with one independent variable. Significance levels were at ^*∗*^*p* < 0.05, ^∗∗^*p* < 0.01, and ^∗∗∗^*p* < 0.001.

## 3. Results

Noncytotoxic ET concentrations that elicit an inflammatory response without affecting cell viability were determined for the different cell lines used in this study. Subsequently, ET was used at 0.1 *µ*g/ml for the treatment of normal mouse mammary epithelial SCp2 cells, at 10 *µ*g/ml for nontumorigenic human mammary epithelial S1 cells and their pretumorigenic counterparts, Cx43-KO S1 cells [21], and at 1 *µ*g/ml for the tumorigenic cell lines including human breast cancer cells of intermediate (MCF-7) and high invasiveness (MDA-MB-231).

### 3.1. Short-Term Treatment with Endotoxin Enhances MMP-9 Levels in Conditioned Media of Normal Mouse (SCp2) and Nontumorigenic Human Mammary Epithelial Cells (S1)

**Figure 1:**
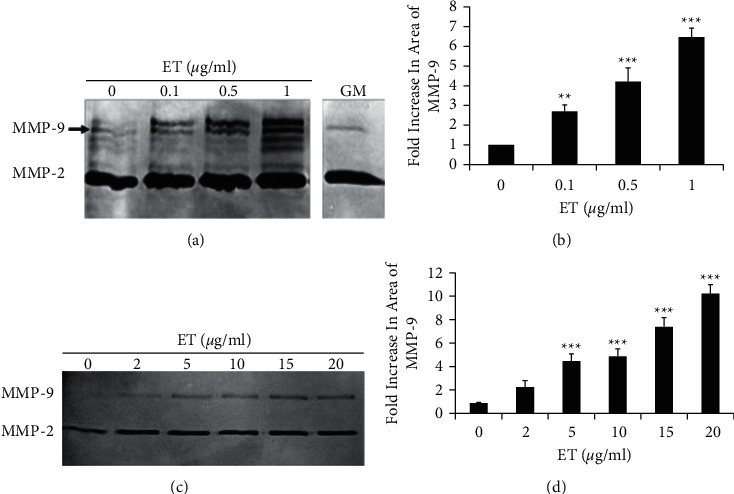
Short-term treatment with endotoxin induces MMP-9 activity in normal mouse (SCp2) and human (S1) mammary epithelial cells. Gelatin zymography of (a) SCp2 and (c) S1 conditioned media collected from untreated control cells (0 µg/ml) and ET-treated cells (0.1, 0.5, and 1 *µ*g/ml for SCp2 cells; 2, 5, 10, 15, and 20 *µ*g/ml for S1 cells) upon reaching 75% confluence after treatment showed dose-dependent upregulation of MMP-9 activity. No differences in the growth profile of ET-treated cells were noted, and all cultures reached 75–80% confluence within the same time frame. The abundant MMP-2 levels noted in the conditioned media of a SCp2 are comparable to basal levels in growth medium (GM). (b, d) Bar graphs represent the quantification of fold increase in peak area of MMP-9 bands shown in the respective zymograms. Each bar represents triplicate analyses of mean ± SD; ^∗∗∗^*P* < 0.001 compared to the untreated control.

SCp2 cells, capable of differentiation upon optimal cell-cell and cell-matrix interactions [22], were treated with 0.1, 0.5, or 1 *µ*g/ml ET for 48 hours (short-term exposure), after which conditioned media were assayed for MMP-9 by zymography. Active MMP-9 levels were upregulated upon treatment with 0.1, 0.5, and 1 *µ*g/ml of ET compared to the untreated control. Quantification of resolved bands denoted ∼2-, 4-, and 6-fold increase in MMP-9 activity in conditioned media of SCp2 cells treated with 0.1, 0.5, and 1 *µ*g/ml of ET, respectively, as compared to the untreated control cells (Figures 1(a) and 1(b)). Similarly, active MMP-9 levels in conditioned media of S1 cells were upregulated upon treatment with ET compared to the untreated control. Quantification of the resolved bands denoted 4.5-, 4.9-, 7.4-, and 10-fold increase in MMP-9 activity in conditioned media of S1 cells treated with 5, 10, 15, and 20 *µ*g/ml of ET, respectively, as compared to the untreated control cells (Figures 1(c) and 1(d)).

### 3.2. Short-Term Exposure to Endotoxin Induces an Inflammatory Response in Human Breast Cancer MDA-MB-231 Cells

**Figure 2:**
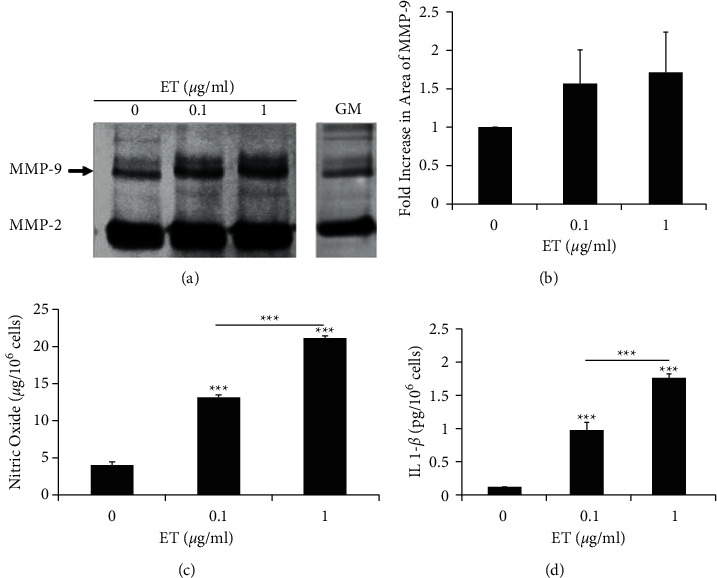
Short-term endotoxin treatment upregulates levels of inflammatory mediators produced by MDA-MB-231 human breast cancer cells. (a) Gelatin zymography of conditioned media collected from untreated control (0 *µ*g/ml) and ET-treated cells (0.1 and 1 *µ*g/ml) upon reaching 75% confluence after treatment showed upregulation of MMP-9 activity in MDA-MB-231 cells. No differences in the growth profile of ET-treated cells were noted, and all cultures reached 75–80% confluence within the same time frame. (b) Quantification of fold increase in peak area of MMP-9 bands shown in the previous zymogram. Each bar represents triplicate analyses of mean ± SD. The abundant unregulated MMP-2 levels noted in the conditioned media are also noted in growth medium (GM). (c) Upregulation of NO and (d) IL 1-*β* levels in MDA-MB-231 conditioned media collected from ET-treated cells (0.1 and 1 *µ*g/ml) compared to untreated controls (0 *µ*g/ml), upon reaching 75% confluence after treatment. Each bar represents triplicate analyses of mean ± SD. ^∗∗∗^*P* < 0.001 compared to the untreated control.

Short-term (48-hour) treatment with either 0.1 or 1 *µ*g/ml ET in tumorigenic MDA-MB-231 human breast cells increased the levels of inflammatory mediators, namely, NO and IL1-*β*. Quantification of the zymography bands denoted an upward, yet non-statistically significant, trend of ∼1.5–1.7-fold increase in MMP-9 activity in conditioned media of MDA-MB-231 cells treated with 0.1 and 1 *µ*g/ml, respectively, as compared to untreated control cells (Figures 2(a) and 2(b)). The levels of NO significantly increased from 3.9 *µ*g/10^6^ cells in conditioned media of control untreated MDA-MB-231 cells to 13.1 *µ*g/10^6^ cells when treated with 0.1 *µ*g/ml ET. A 5-fold increase in NO level (20 *µ*g/10^6^ cells) was noted when treated with 1 *µ*g/ml ET (Figure 2(c)). Levels of IL1-*β* increased from 0.1 pg/10^6^ cells in conditioned media of untreated cells to 0.9 pg/10^6^ cells when treated with 0.1 *µ*g/ml ET. Cells treated with 1 *µ*g/ml ET had an 18-fold increase (1.8 pg/10^6^ cells) in their conditioned media compared to untreated controls (Figure 2(d)).

### 3.3. Long-Term, but Not Short-Term, Endotoxin Treatment Enhances Migration of Normal Mouse Mammary Epithelial Cells (SCp2) and Human Breast Cancer Cells (MCF-7 and MDA-MB-231)

**Figure 3:**
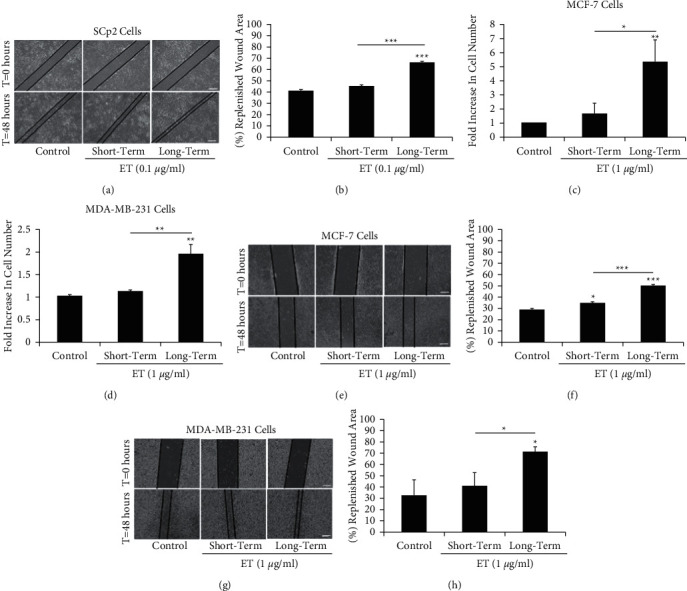
Long-term endotoxin treatment enhances migration of normal mouse mammary epithelial cells (SCp2) and human breast cancer cells (MCF-7 and MDA-MB-231). (a) Wound healing assay showed enhanced replenishment of wound area with migrating SCp2 cells upon long-term exposure (for one month) to 0.1 *µ*g/ml ET, as opposed to short-term exposure (for 48 hours after wounding) or untreated controls (0 *µ*g/ml). (b) Bar graph shows the percentage of the replenished wound area with migrating SCp2 cells upon short-term and long-term exposure to 0.1 *µ*g/ml ET relative to untreated controls, for 48 hours after wounding. (c) Long-term exposure to 1 *µ*g/ml ET increased the proliferation rate of MCF-7 and (d) MDA-MB-231 cells, as opposed to short-term exposure, where the increase in cell count was not significant when compared to untreated controls (0 *µ*g/ml). Wound healing assay showed enhanced migration of (e, f) MCF-7 upon short (albeit marginal) and long-term exposures and (g, h) MDA-MB-231 upon long-term exposure to 1 *µ*g/ml ET, when compared to untreated controls (0 *µ*g/ml). Scale bar = 100 *µ*m. Each bar represents triplicate analyses of mean ± SD. ^*∗*^*P* < 0.05; ^∗∗^*P* < 0.01; ^∗∗∗^*P* < 0.001 compared to the untreated control.

Wound healing assay showed that the wounded area noted in the short-term treated SCp2 cells was not significantly different when compared to untreated controls. However, long-term exposure to 0.1 *µ*g/ml ET enhanced the closure of the wound, whereby 66% of the wounded area was repopulated with migrating cells, compared to only 41% in untreated controls 48 hours after wounding (Figures 3(a) and 3(b)). After noting the response of nontumorigenic mammary cells to ET exposure, we opted to investigate the effect of ET treatment (1 *µ*g/ml) on modulating migration and proliferation of human breast cancer low invasive MCF-7 and highly invasive MDA-MB-231 cells. Interestingly, long-term, but not short-term, exposure to 1 *µ*g/ml ET enhanced the proliferation rate of MCF-7 and MDA-MB-231 cells. Short-term ET treatment did not significantly enhance the proliferation rate, while the number of cells exposed to long-term ET treatment was ∼5 and 1.9 times greater than that of untreated MCF-7 (Figure 3(c)) and MDA-MB-231 cells (Figure 3(d)), respectively. In MCF-7 cells, short-term exposure to 1 *µ*g/ml ET had a marginal, yet significant, effect while long-term exposure to 1 *µ*g/ml ET markedly enhanced the closure of the wound compared to the untreated control; 50% of the wounded area was repopulated with migrating cells, compared to only 28% in untreated controls at 48 hours after wounding (Figures 3(e) and 3(f)). On the other hand, closure of the wounded area noted in the short-term treated MDA-MB-231 cells was not significantly different when compared to the untreated control; however, long-term exposure to 1 *µ*g/ml ET enhanced the closure of the wound compared to untreated controls, whereby 72% of the wounded area was repopulated with migrating cells, compared to only 33% in untreated controls at 48 hours after wounding (Figures 3(g) and 3(h)). It is worth noting that long-term treatment of ET on low invasive MCF-7 cells had a more pronounced effect on enhancing proliferation (5-fold) than invasion (1.6-fold), whereas ET treatment of highly invasive MDA-MB-231 cells equally enhanced (2-fold) migration and proliferation.

### 3.4. Endotoxin Preferentially Enhances Invasion in Pretumorigenic Human Breast Epithelial Cx43-KO S1 Cells

**Figure 4:**
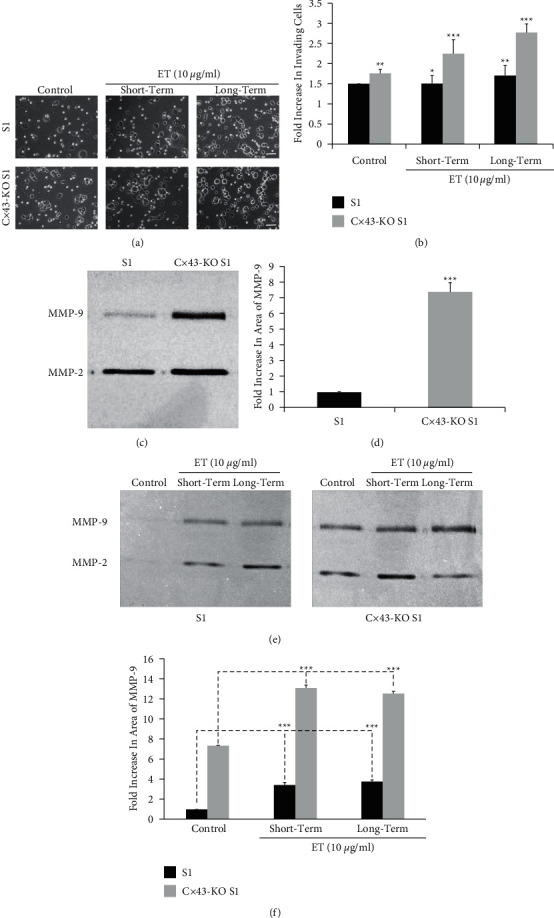
Pretumorigenic Cx43-KO S1 human breast epithelial cells secrete higher levels of MMP-9, and their invasive ability is enhanced upon endotoxin exposure more than their nontumorigenic S1 counterparts. (a) Short-term and long-term exposures of S1 and Cx43-KO S1 cells to 10 *µ*g/ml ET enhanced the invasion across Matrigel. Scale bar is 10 *µ*m. (b) Fold increase in the number of invading cells subjected to short-term and long-term ET treatment at 10 *µ*g/ml ET relative to untreated controls (0 *µ*g/ml). (c) Gelatin zymography of untreated S1 and Cx43-KO S1 media collected from 2D cultures upon reaching 75% confluence showed upregulation of MMP-9 activity in Cx43-KO S1 compared to S1 cells. (d) Quantification of fold increase in peak area of MMP-9 bands shown in the previous zymogram, corrected for the 30% increase in cell counts observed with Cx43-KO S1 compared to S1 cells. (e) Gelatin zymography and (f) quantification of fold increase in peak area of MMP-9 bands of conditioned media collected from 2D cultures of S1 and Cx43-KO S1 cells, including untreated controls (0 *µ*g/ml) and ET-treated (10 *µ*g/ml) cells, upon reaching 75% confluence after treatment and after long-term exposure (one month), corrected for the 30% increase in cell counts of Cx43-KO S1 compared to S1 cells. Results showed upregulation of MMP-9 activity in S1 and Cx43-KO S1 culture media after both ET exposures, as compared to untreated controls of each cell type. Samples of each cell type were run on the same gel and under the same conditions. Each bar represents triplicate analyses of mean ± SD. ^*∗*^*P* < 0.05; ^∗∗^*P* < 0.01; ^∗∗∗^*P* < 0.001 compared to the untreated control.

Recent studies in our laboratory showed that the loss of expression of the gap junction protein connexin 43 (Cx43) triggered cell cycle entry and invasion through basement membrane in the nontumorigenic human breast epithelial S1 cells [19]. To determine the effect of ET treatment on invasion, transwell cell invasion assay was performed. Untreated Cx43-KO S1 cells acquired ∼1.8-fold increase in their invasion capacity compared to control untreated S1 cells (Figures 4(a) and 4(b)). This enhanced invasion capacity was consistent with previous studies in our laboratory [19]. Short-term (9-day) and long-term (one-month) ET treatment of S1 cells induced around 1.5-fold increase in the invasion capacity of S1 cells, compared to control untreated cells (Figure 4(b)). Interestingly, Cx43-KO S1 cells subjected to short-term ET treatment showed ∼2.2-fold increase in their ability to invade through the Matrigel, while the long-term exposure led to ∼2.8-fold increase in the invasion capacity of ET-treated Cx43-KO S1 cells, compared to control untreated S1 cells (Figure 4(b)).

It is noteworthy that the activity levels of basal MMP-9 secreted by Cx43-KO S1 were higher compared to those of S1 cells. Cx43-KO S1 cells exhibit a faster rate of proliferation [19]. Thus, correcting for a 30% difference in cell numbers noted in Cx43-KO S1 compared to S1 cells, we detected a 7-fold increase in MMP-9 activity in Cx43-KO S1 medium as compared to that of S1 cells (Figures 4(c) and 4(d)). In addition, Cx43-KO S1 cells consistently maintained higher levels of MMP-9 activity, compared to S1 cells upon short-term and long-term ET treatment (Figures 4(e) and 4(f)).

### 3.5. Endotoxin Treatment Disrupts Lumen Formation in Nontumorigenic Human Breast Epithelial S1 Acini

**Figure 5:**
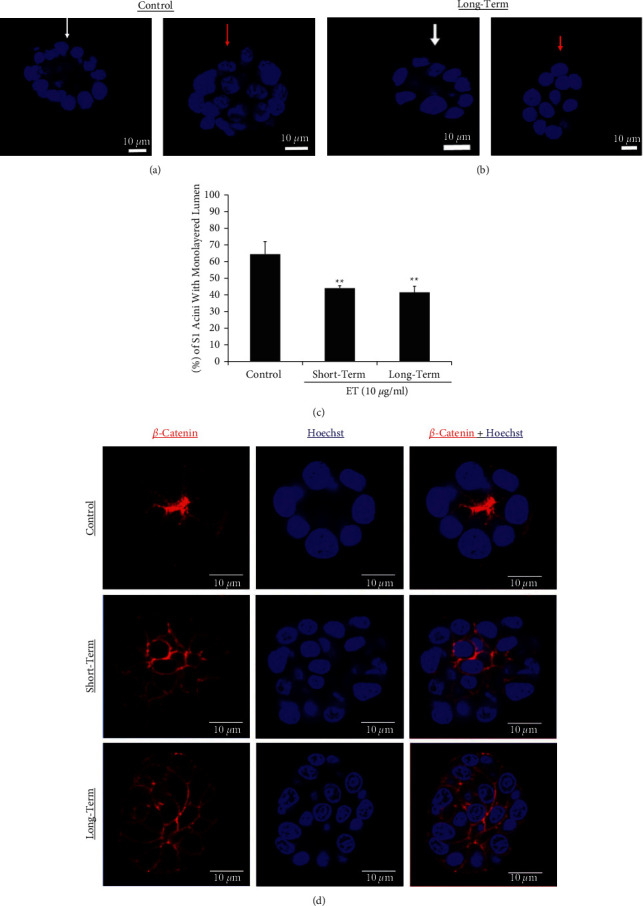
Endotoxin disrupts lumen formation in 3D cultures of human breast epithelial S1 cells. Acini of (a) control untreated S1 cells (0 *μ*g/ml) and (b) S1 cells subjected to long-term ET treatment at 10 *μ*g/ml were stained with Hoechst (blue) on day 11 and scored for lumen formation. White arrows point at acini with normal monolayered lumen, while red arrows point at those with disrupted multilayered lumen. (c) Bar graph shows percentages of acini with undisrupted monolayered lumen in control untreated S1 cells and those subjected to short-term as well as long-term ET treatment at 10 *μ*g/ml. One hundred acini were scored for each condition in every replicate. Each bar represents triplicate analyses of mean ± SD. ^∗∗^*P* < 0.01 compared to the untreated control. (d) Representative S1 acini immunostained for *β*-catenin (red) and counterstained with Hoechst (blue). The upper lane shows a control untreated S1 acinus with undisrupted monolayered lumen and apicolateral *β*-catenin localization. The middle lane shows an S1 acinus following short-term ET treatment at 10 *μ*g/ml, and the lower lane shows an S1 acinus following long-term ET treatment at 10 *μ*g/ml. Both short-term and long-term treatments reveal higher abundance of multilayered acini devoid of lumen, with relocalization of *β*-catenin across the entire cell membrane.

S1 cells in 3D cultures organize into differentiated monolayer acini surrounding a lumen, with a well-established apical polarity [23]. We have previously demonstrated that the loss of Cx43 expression disrupts this S1 cell normal acinar morphology and epithelial cell polarity [21]. To determine whether ET treatment could influence the organization of the breast epithelium, S1 cells were subjected to short-term and long-term treatment, after which acinar morphogenesis and lumen formation were assessed. While 64% of the control untreated S1 acini displayed typical lumen structures enclosed within a single layer of cells (Figure 5(a) control and 5(d) control), only 40–45% of the S1 acini treated for short and long periods with 10 *µ*g/ml ET had normal morphology with undisrupted lumen demonstrating that ET treatment disrupted acinar morphology (Figures 5(b) and 5(c)).

### 3.6. Endotoxin Treatment Disrupts *β*-Catenin Localization in S1 Acini

**Figure 6:**
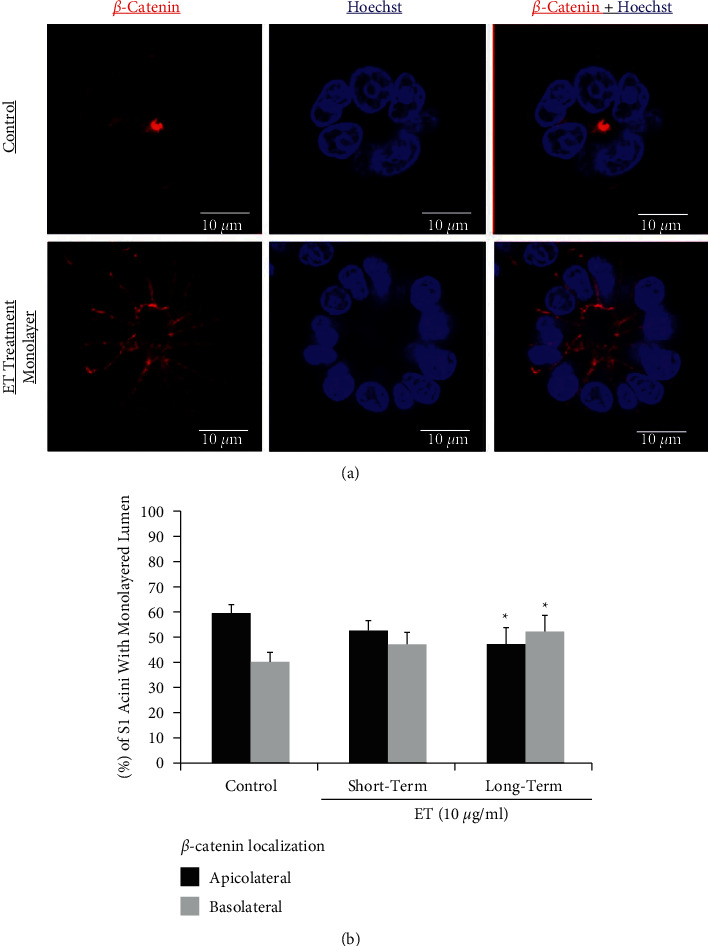
Long-term endotoxin treatment alters *β*-catenin localization in 3D cultures of human breast epithelial S1 cells. (a) Representative S1 acini immunostained for *β*-catenin (red) and counterstained with Hoechst (blue). The upper lane shows a control untreated S1 acinus with undisrupted monolayered lumen and apicolateral *β*-catenin localization. The lower lane shows an S1 acinus following ET treatment at 10 *μ*g/ml revealing relocalization of *β*-catenin from the apicolateral to the lateral and basolateral domain while maintaining undisrupted monolayered lumen. (b) Quantification data of *β*-catenin localization shows significant redistribution of *β*-catenin from the apicolateral to the basolateral domain in S1 acini with monolayered lumen after long-term ET treatment at 10 *μ*g/ml. Localization of *β*-catenin was evaluated under confocal microscopy. One hundred acini with monolayered lumen from every replicate were visualized, and each acinus was scored for polarity based on the *β*-catenin (red) localization as apicolateral versus basolateral. Each bar represents triplicate analyses of mean ± SD. ^*∗*^*P* < 0.05 compared to the untreated control.

Data from our laboratory has shown that S1 acini with correct morphology display apicolateral distribution of *β*-catenin and gap junctional complexes [21] (Figure 5(d) control and 6(a) control). Consequently, we determined whether ET treatment would induce *β*-catenin mislocalization in S1 acini. This indeed was noted in acini with disrupted lumen, whether in control untreated (data not shown) or short-term or long-term ET-treated acini, whereby *β*-catenin was redistributed across the entire cell membrane (Figure 5(d)). Interestingly, among the S1 acini with monolayered lumen in the control group (Figure 6(a)), ∼60% showed apicolateral localization of *β*-catenin (Figure 6(a) top lane), while the remaining ∼40% had more prominent lateral and basal redistribution of *β*-catenin (Figure 6(b)). This ratio was not significantly altered following short-term exposure to ET; however, upon long-term ET exposure, only ∼48% of the S1 acini with monolayered lumen maintained apicolateral localization of *β*-catenin, while those with more prominent lateral and basal redistribution increased to ∼52% (Figure 6(b)).

## 4. Discussion

Despite the presence of previous research associating chronic inflammation with malignant transformation in many tissues [15], little is known about the effect of inflammation on breast cancer initiation or progression. Whether inflammation might trigger tumor initiation events, noted by loss of normal morphological features of mammary epithelium, cell cycle entry, and enhanced migration and invasion [21], is not well known. Our study investigates the role of ET-induced inflammatory insult in breast cancer initiation events using nontumorigenic (SCp2 and S1) rodent mammary and human breast epithelial cells, respectively, and whether such an insult can “add injury” to pretumorigenic (Cx43-KO S1) or tumorigenic breast cells (MCF-7 and MDA-MB-231).

### 4.1. Inflammation and Breast Cancer

The use of ET in in vitro and in vivo models to simulate inflammation-like conditions is widely accepted. In vitro, ET was shown to induce inflammatory phenotype in alveolar epithelial cells [24], umbilical vein endothelial cells [25], and bovine and rodent mammary epithelial cells [18, 26, 27], in addition to stimulating macrophages [28, 29], T cells [30], and B cells [31] from different species. In vivo, ET induced mastitis in several animal models, when introduced into the mammary gland of sheep [32], goat [33, 34], and cows [35, 36].

In line with our current findings, previous studies showed that ET exposure modulated the function of both normal and tumorigenic cells. ET inhibited the expression of the differentiation marker *β*-casein, activated NF-*κ*B, increased gelatinase activity, and stimulated the production of NO and inflammatory cytokines, such as IL-6 and tumor necrosis factor *α* (TNF-*α*), in nontumorigenic mouse mammary SCp2 and CID-9 cells [17, 18]. In tumorigenic cells, ET induced tumor angiogenesis via increased IL-6 and VEGF production by stromal fibroblasts isolated from colon cancer [37] and enhanced lung metastasis in a xenograft mouse model [38]. Moreover, plasma collected from ET-stimulated human and rodent blood was shown to activate the NF-*κ*B pathway and to enhance migration of prostate cancer cells in vitro [11].

Long-term ET treatment augmented proliferation and migration of moderately (MCF-7) and highly invasive human breast cancer cells (MDA-MB-231), suggesting that inflammation enhances tumorigenicity of breast cancer cells. Indeed, inflammation has been reported to accelerate tumor progression in mouse models of breast cancer (reviewed by [39]). Moreover, chemotherapy-induced inflammation was reported as a main contributor to chemoresistance and metastasis in both syngeneic and xenograft breast cancer models [40]. Trivanović et al. demonstrated that MCF-7 cells acquire epithelial-to-mesenchymal transition (EMT) properties after exposure to conditioned media collected from inflammation-primed human adipose cells [41]. Similarly, Hong et al. demonstrated that ET treatment induces an EMT-like phenotype in MDA-MB-231 cells, leading to enhanced migration and invasion [42]. Our results are further supported by the previously proposed mechanism involving Toll-like receptor 4 (TLR4), overexpressed in breast cancer patients with lymph node metastasis, that was induced in both ET-treated human breast cancer cells, MCF-7 and MDA-MB-231, resulting in increased invasiveness [43]. Indeed, TLR4 induces the expression of T-LAK cell-Originated Protein Kinase (TOPK) that has been proposed to regulate MMP-9 gelatinase expression and activity, thus acting as key mediator of ET-induced migration and invasion of human breast adenocarcinoma cell lines [44].

### 4.2. Inflammatory Microenvironment and Cell Phenotype

The cellular microenvironment plays a major role in transitioning into a transformed phenotype [45]. Specifically, an inflammatory insult, which enhances the expression of cytokines and MMPs, may induce dedifferentiation and loss of normal tissue phenotype, an early sign of breast cancer [46, 47]. It is noteworthy that obesity was shown to trigger breast adipose tissue inflammation, leading to increased risk of breast cancer [48, 49], and has been associated with loss of the apical distribution of Cx43 in 3D cultures of S1 breast epithelial cells, mitotic spindle orientation (MSO), and induced proliferation and multilayering in S1 acini [19, 21]. This oncogenic effect is believed to be mediated by the fat tissue-derived adipokine leptin [50, 51]. Interestingly, we demonstrated that ET treatment of S1 acini impaired the normal morphogenesis, as indicated by the disrupted formation of monolayered lumen in 3D cultures of S1 cells and the altered localization of *β*-catenin. Our lab has previously shown that the loss of Cx43 expression in S1 cells triggers cell cycle entry and invasion through basement membrane [19, 21]. While ET treatment upregulated the activity of MMP-9 and concomitantly enhanced the invasion of S1 cells, those effects were more pronounced in their Cx43-KO S1 pretumorigenic counterparts. This suggests that pretumorigenic breast cells are more sensitive to ET insults than normal cells and indicates that an inflammatory microenvironment adds injury to tumor-initiated cells. An earlier study by Riccardi et al. highlighted the effect of inflammation on cancer progression, whereby neoplastic epithelial cells acquired a mesenchymal phenotype through inflammatory stimuli. The mesenchymal phenotype was manifested as immune-regulatory functions and other immune-inhibitory properties, typically expressed by mesenchymal-stromal cells, which lead to tumor immune escape and cancer progression [52].

## 5. Conclusion

In conclusion, this study shows that ET-induced inflammation triggers or enhances tumor initiation events in nontumorigenic and pretumorigenic breast epithelia, respectively, and enhances tumorigenicity of breast cancer cells. Our findings highlight the role of inflammation in inducing cancer initiation events, portrayed as loss of normal differentiated morphology in 3D cultures of nontumorigenic breast cells, in addition to increasing the migratory and invasive abilities. Our study also suggests that such inflammatory insults can “add injury” to pretumorigenic and tumorigenic breast cells. Future studies should focus on studying the tumor-initiating ability of ET-treated Cx43-KO S1 cells, and the tumor-enhancing ability of ET-treated MDA-MB-231 and MCF-7 cells in vivo as compared to their untreated counterparts.

## Data Availability

Data sharing is not applicable to this article as no datasets were generated or analyzed during the current study.
